# Annual acknowledgement of reviewers

**DOI:** 10.1186/1471-2350-14-22

**Published:** 2013-03-07

**Authors:** Tim Sands

**Affiliations:** 1 BioMed Central, Floor 6, 236 Gray's Inn Road, London WC1X 8HB, UK

## Abstract

**Contributing reviewers:**

The editors of BMC Medical Genetics would like to thank all our reviewers who have contributed to the journal in Volume 13 (2012).

## 

Ryan Abo

USA

Adebowale Adeyemo

USA

Ilir Agalliu

USA

Atif Ahmed

USA

Guruprasad Aithal

UK

Eleni Aklillu

Sweden

Elizabeth Algar

Australia

Bassam Ali

United Arab Emirates

Rando Allikmets

USA

Richard Anney

Ireland

Muhammad Ansar

Pakistan

Marcello Arca

Italy

Philip Asherson

UK

Valeria Bafunno

Italy

Pantelis Bagos

Greece

Melanie Bahlo

Australia

Siddharth Banka

UK

Michael Barnes

UK

Cristina Basso

Italy

Veronique Bataille

UK

Gareth Baynam

Australia

Britt-Maria Beckmann

Germany

Elijah Behr

UK

Evangelos Bellos

UK

Lorenzo Beretta

Italy

Arthur Bergen

Netherlands

Andreia Bettencourt

Portugal

Connie Bezzina

Netherlands

M.Z.A. Bhuiyan

Switzerland

Louise Bicknell

UK

Ayşe Bilgiç

Turkey

Firouzeh Biramijamal

Iran

Kathryn Blake

USA

Luigi Boccuto

USA

Amelie Bonnefond

France

Paulina Borkowska

Poland

Mridula Bose

India

Yohan Bosse

Canada

Yvonne Böttcher

Germany

Nicoletta Botto

Italy

Nabila Bouatia-Naji

France

Kym Boycott

Canada

Francesco Brancati

Italy

Gerome Breen

UK

John Brehm

USA

Paul Breslin

USA

Nicola Brunetti-Pierri

Italy

Damien Bruno

Australia

William Bruno

Italy

Helene Bruyere

Canada

David Bunyan

UK

Adam Butterworth

UK

Miguel Calero

Spain

Lisa Cameron

Canada

Vicky Cameron

New Zealand

Craig Campbell

Canada

Natalia Cannelli

Italy

Carlos Cano Gutierrez

Spain

Federico Canzian

Germany

Wangsen Cao

China

Yunxia Cao

China

Valeria Capra

Italy

Elena Cardaioli

Italy

Suzan Carmichael

USA

Luis Carvajal Carmona

UK

Agostinho Carvalho

Italy

Sergi Castellvi-Bel

Spain

Marco Castori

Italy

Gianpiero Cavalleri

Ireland

Angelo Baldassare Cefalu

Italy

Peter Celec

Slovakia

Marie Cerna

Czech Republic

Stormy Chamberlain

USA

Giriraj Chandak

India

Sybil Charrière

France

Chih-Ping Chen

Taiwan

Suet Nee Chen

USA

Zhuo Chen

USA

Yu-Ching Cheng

USA

Bernard Cheung

Hong Kong

Pietro Chiurazzi

Italy

Young Min Choi

Korea, South

Jieun Choi

Korea, South

Helene Choquet

USA

Imke Christiaans

Netherlands

Vincent Christoffels

Netherlands

Denise Christofolini

Brazil

Shu-Chun Chuang

UK

Paola Ciotti

Italy

Jacqueline Cloos

Netherlands

Dawn Coletta

USA

Oscar Cordero

Spain

Victoria Cortessis

USA

Helena Cortez-Pinto

Portugal

Stefania Corti

Italy

Sara Courtneidge

USA

Damien Croteau-Chonka

USA

Eugenia Cruz

Portugal

Daniel Cyr

Canada

Thiago da Silva

Brazil

Min Dai

China

Denise Daley

USA

GM Dallinga-Thie

Netherlands

Collet Dandara

South Africa

Saeed Daneshmandi

Iran

Paul de Bakker

USA

Lawrence de Koning

USA

Tim De Meyer

Belgium

Claudia Vitoria De Moura Gallo

Brazil

Marina De Rosa

Italy

Maria de Sousa

Portugal

Matt Deardorff

USA

George Dedoussis

Greece

Abbas Dehghan

Netherlands

Ranjan Deka

USA

Jesús Delgado Calle

Spain

Brian Delisle

UK

Esteban Dell'Angelica

USA

Julie Demars

France

Hao Deng

USA

Rozalia Dimitriou

UK

James Dowling

USA

Ilias Doxiadis

Netherlands

Jubao Duan

USA

Thomas Eggermann

Germany

Stefanie Eggers

Australia

Philipp Ehlermann

Germany

Elisabet Einarsdottir

Finland

Laure Elens

Belgium

Nathan Ellis

USA

Julia El-Sayed Moustafa

UK

Matthias Elstner

Germany

Charlotta Enerback

Sweden

James Engert

Canada

Emel Ergul

Turkey

Héctor Escobar-Morreale

Spain

Gareth Evans

UK

Larissa Fabritz

Germany

Yi-Ming Fan

China

Silvia Fargion

Italy

Paula Faustino

Portugal

Martha Felini

USA

Jane Ferguson

USA

Agustin Fernandez

Spain

Carlos Flores

Spain

Erick Forno

USA

Rachel Freathy

UK

Robert Fredriksson

Sweden

Ana Freitas

Portugal

Jan Friedman

Canada

Mike Friez

USA

Jennifer Frost

USA

Maria Fuciarelli

Italy

Tibor Fulop

USA

Frédéric Fumeron

France

Frédéric Galacteros

France

Louise Gallagher

Ireland

Elena Garcia-Martin

Spain

Valentina Gatta

Italy

Massimo Gennarelli

Italy

Maurizio Genuardi

Italy

Manuela Germeshausen

Germany

Guillermo Gervasini

Spain

Uday Ghoshal

India

Todd Gibson

USA

Hendrika Ginjaar

Netherlands

David Godler

Australia

Kwang-Il Goh

Korea, South

Marilda Goncalves

Brazil

Maria Gonsebatt

Mexico

Paola Grammatico

Italy

Struan Grant

USA

Julia Greer

USA

Dongfeng Gu

China

Regina Maria Guaragna

Brazil

Daniel Guerrier

France

Mingzhou Guo

China

Xiong Guo

China

Zhongmin Guo

USA

Christina Gurnett

USA

Fiorella Gurrieri

Italy

Nancy Hadley Miller

USA

Won-Ho Hahn

South Korea

D. Michael Hallman

USA

Jiali Han

USA

Dana Hancock

USA

Torben Hansen

Denmark

Sarah Harten

Australia

Qurratulain Hasan

India

Mohammad Hashemi

Iran

Ziarih Hawi

Australia

Ping-An He

China

Xiang-Ge He

China

James Hejtmancik

USA

Jose Hernandez

Spain

Concepción Hernández-Chico

Spain

Nattiya Hirankarn

Thailand

James Hixson

USA

Gunter Hoglinger

Germany

Laura Horsfall

UK

Kikuko Hotta

Japan

James Howe

USA

Chuen Hsueh

Taiwan

Zhibin Hu

China

Maria Hughes

UK

Chia-Cheng Hung

Taiwan

Maria Hurle

Spain

Mohammad Ilyas

UK

Ken Inoue

Japan

Ituro Inoue

Japan

Iuliana Ionita-Laza

USA

Heikki Jarvinen

Finland

Yu-Wu Jiang

China

Philippe Joly

France

Erik Jönsson

Sweden

Suh-Hang Juo

Taiwan

Bernadette Kalman

Germany

Stavroula Kanoni

UK

Tom Karlsen

Norway

Tadafumi Kato

Japan

Tomohiro Katsuya

Japan

Xiayi Ke

UK

Dagmar Keller

Switzerland

Chiea Khor

Singapore

Madhu Khullar

India

Stefan Kiechl

Austria

Akinori Kimura

Japan

Youlia Kirova

France

Krzysztof Kiryluk

USA

Robert Kiss

Canada

Hiroaki Kitaoka

Japan

Rick Kittles

USA

Stanislav Kmoch

Czech Republic

Julian Knight

UK

Barbara Kofler

Austria

Haris Kokotas

Greece

Yoshihiko Kominato

Japan

Tamara Koopmann

Canada

Peter Kovacs

Germany

Ian Krantz

USA

Marcin Krawczyk

Germany

David Kreil

Austria

Christos Kroupis

Greece

Takeo Kubota

Japan

Aya Kuchiba

USA

Jitender Kumar

Sweden

Fuat Kurbanov

USA

Mateusz Kurzawski

Poland

Nan Laird

USA

Jatinder Lamba

USA

Pier Lambiase

UK

Xun Lan

USA

Joung-Liang Lan

Taiwan

Tanja Langsenlehner

Austria

Matthew Lanktree

Canada

Catherine Laprise

Canada

Bernard Le Foll

Canada

Solena Le Scouarnec

UK

Cecile Lecoeure

France

Pauline Lee

USA

Mikko Lehtovirta

Finland

Marianne Leruez-Ville

France

Gaetan Lesca

France

Esther Leshinsky-Silver

Israel

Ben Li

USA

Kuanyu Li

China

Jialiang Li

Singapore

John Lima

USA

Cheng-Chieh Lin

Taiwan

Javier Llorca

Spain

Paul Lochhead

USA

Katja Lohmann

Germany

Marie-Anne Loriot

France

Fengmin Lu

China

Michelle Luciano

UK

Hung Luu

USA

Ikuhiro Maeda

Japan

Shiro Maeda

Japan

Shingo Maeda

Japan

Jane Maguire

Australia

Nejat Mahdieh

Iran

Nerea Maiz

Spain

Carmen Maldonado-Bernal

Mexico

Giovanni Malerba

Italy

Ayse Tulin Mansur

Turkey

Agustin Martinez

Chile

Tara Matise

USA

Ross McManus

Ireland

Kim McBride

USA

Thomas McDonald

USA

Ken McElreavey

France

Pascal McKeown

UK

Benjamin Meder

Germany

Carolina Medina-Gomez

Netherlands

Andre Megarbane

Lebanon

Lorenzo Melchor

UK

George Mellick

Australia

Ludwine Messiaen

USA

David Meyre

Canada

Maria Giuseppina Miano

Italy

Anne Marie Minihane

UK

Monica Miozzo

Italy

Masatomo Miura

Japan

Jamal Mohamed

USA

Pierre Morange

France

Rodrigo Moreno-Reyes

Belgium

Derek Morris

Ireland

Richard Myers

USA

Alessio Naccarati

Italy

Muhammad Naeem

Pakistan

Ramaswamy Narayanan

USA

Mark Nellist

Netherlands

Grethe Neumann Andersen

Denmark

Finn Cilius Nielsen

Denmark

Stefano Nobile

Italy

Ann Nordgren

Sweden

Margareta Nordling

Sweden

Boris Novakovic

Australia

William Oetting

USA

Kenji Okumura

Japan

Shoichiro Ono

USA

Patrick Onyango

USA

Antonio Orlacchio

Italy

Kayo Osawa

Japan

Tadayuki Oshima

Japan

Andy Overall

UK

Nicolas Pallet

France

Dimitrios Papazoglou

Greece

Jillian Parboosingh

Canada

Hemang Parikh

USA

Hae-Sim Park

South Korea

Vickas Patel

USA

Edyta Pawlak-Adamska

Poland

Palle Lyngsie Pedersen

Denmark

Jose-Luis Perez-Castrillon

Spain

Pablo Perez-Martinez

Spain

Inga Peter

USA

Ruth Pfeiffer

USA

Catherine Phillips

Ireland

Marie Pigeyre

France

Julia Pinsonneault

USA

Carlos Pirola

Argentina

Pyotr Platonov

Sweden

Rafal Ploski

Poland

Alex Postma

Netherlands

Lincoln Potter

USA

Christian Prinz

Germany

Susana Puig

Spain

Qibin Qi

USA

Hui-Qi Qu

USA

Luis Quiñones

Chile

Mihaescu Raluca

Netherlands

Luca Rampoldi

Italy

Michele Ramsay

South Africa

Deepa Selvi Rani

India

Roberto Ravazzolo

Italy

Umbertina Reed

Brazil

Ralf Reilmann

Germany

Fulvio Ricceri

Italy

Michael Rieder

Canada

Gonzalo Rincon

USA

William Rizzo

USA

Kathryn Robson

UK

Jacques Rochette

France

Angela Roco

Chile

Benjamin Rodriguez

USA

Laia Rodriguez-Revenga

Spain

Manuel Romero-Gomez

Spain

Konstantinos Rouskas

Greece

Giuseppina Russo

Italy

Brid Ryan

USA

Euijung Ryu

USA

Matthew Sabin

Australia

Norihisa Saeki

Japan

Richard Saffery

Australia

Takeyori Saheki

Japan

Zainab Samaan

Canada

Aga Syed Sameer

India

Mark Samuels

Canada

Valeria Sandrim

Brazil

Federica Sangiuolo

Italy

Filippo Santorelli

Italy

Nicola Santoro

USA

José Antonio Sainz 

Spain

José Santos

Chile

Daimei Sasayama

Japan

Shizuka Sasazuki

Japan

Shigeru Satoh

Japan

Jeremiah Scharf

USA

Nicolas Schröder

Germany

Brian Schutte

USA

Michal-Ruth Schweiger

Germany

Ronnie Sebro

USA

Shantanu Sengupta

India

Michael Shapero

USA

Manu Sharma

Germany

Joseph Shieh

USA

Beverley Shields

UK

Vorasuk Shotelersuk

Thailand

Hyagriv Simhan

USA

Alessandro Simonati

Italy

Benedetto Simone

Italy

Helen Skaletsky

USA

Rebecca Slager

USA

Anne Slavotinek

USA

Caren Smith

USA

Jonathan Smith

USA

Katie Smith

UK

Qing Song

USA

José Luis Soto

Spain

Alessio Squassina

Italy

Horia Stanescu

UK

Frauke Stanke

Germany

Boban Stanojevic

Serbia

Robert Steiner

USA

Elena Sticchi

Italy

Catherine Stolle

USA

Patrick Stover

USA

Tomoko Sugiura

Japan

Jielin Sun

USA

Menha Swellam

Egypt

Jeff Swensen

USA

Csaba Szalai

Hungary

Dar-In Tai

Taiwan

Mayumi Tamari

Japan

Wenru Tang

China

Nelson Tang

Hong Kong

Kelan Tantisira

USA

Muhammad Tariq

USA

Kumarasamy Thangaraj

India

Mads Thomassen

Denmark

Francesco Danilo Tiziano

Italy

Jun Tohyama

Japan

Amanda Toland

USA

Rossella Tricarico

Italy

Tom Trikalinos

USA

Jaroslav Truksa

Czech Republic

Stephen Tsang

USA

Daniela Turchetti

Italy

Benjamin Tycko

USA

Ching-Cherng Tzeng

Taiwan

Karen Usdin

USA

Luca Valenti

Italy

Maarten van den Berge

Netherlands

Paul van Diest

Netherlands

Marie van Dijk

Netherlands

Geert van Loo

Belgium

Sakari Vanharanta

USA

Francis Vasseur

France

Thirumalaisamy Velavan

Germany

Gilberto Velho

France

Kabilan Velliyagounder

USA

Sebastien Viatte

UK

Anxo Vidal

Spain

Laurent Villard

France

Chris Wallace

UK

Xingyu Wang

China

Yao Wang

USA

Jingbo Wang

Singapore

Yong Wang

China

Junbai Wang

Norway

Christina Wassel

USA

Gerald Watts

Australia

Mary Waye

Hong Kong

Kate Weiner

UK

Bo Wen

China

Patrick Weydt

Germany

Scott Williams

USA

Katharina Wimmer

Austria

Robert Winqvist

Finland

Katarzyna Wozniak

Poland

Lei Xiao

USA

Hongyan Xu

USA

Ya-Wei Xu

Colombia

Norishige Yamada

USA

Ian Yang

Australia

Mao Sheng Yang

China

Akihiro Yasoda

Japan

Dong-Qing Ye

China

Serbulent Yigit

Turkey

Ming-Whei Yu

Taiwan

Chenggong Yu

USA

Wenqiang Yu

China

Jin-Tai Yu

China

Pierre Zalloua

Lebanon

Kevin Zbuk

Canada

Tanja Zeller

Germany

Zheng Zeng

China

Yun Zhang

UK

Lin Zhang

China

Yujing Zhang

USA

Xiangtian Zhou

China

Huiping Zhu

USA

Michael Zimmer

Germany

Sarah Zimmerman

USA

Elias Zintzaras

Greece

Robert Zivadinov

USA

Gabor Zsurka

Germany

Christiane Zweier

Germany

## Pre-publication history

The pre-publication history for this paper can be accessed here:

http://www.biomedcentral.com/1471-2350/14/22/prepub

